# Arachidonic acid‐derived dihydroxy fatty acids in neonatal cord blood relate symptoms of autism spectrum disorders and social adaptive functioning: Hamamatsu Birth Cohort for Mothers and Children (HBC Study)

**DOI:** 10.1111/pcn.13710

**Published:** 2024-07-23

**Authors:** Takaharu Hirai, Naoko Umeda, Taeko Harada, Akemi Okumura, Chikako Nakayasu, Takayo Ohto‐Nakanishi, Kenji J. Tsuchiya, Tomoko Nishimura, Hideo Matsuzaki

**Affiliations:** ^1^ Department of Psychiatric and Mental Health Nursing, School of Nursing University of Fukui Eiheiji Japan; ^2^ Life Science Innovation Center University of Fukui Eiheiji Japan; ^3^ Department of Maternal and Child Health Nursing, School of Nursing University of Fukui Eiheiji Japan; ^4^ Research Center for Child Mental Development Hamamatsu University School of Medicine Hamamatsu Japan; ^5^ United Graduate School of Child Development, Osaka University, Kanazawa University, Hamamatsu University School of Medicine Chiba University and University of Fukui Suita Japan; ^6^ Lipidome Lab Co., Ltd. Akita Japan; ^7^ Research Center for Child Mental Development University of Fukui Eiheiji Japan

**Keywords:** adaptive functioning, arachidonic acid, autism, cord blood, dihydroxy eicosatetraenoic acid

## Abstract

**Aim:**

Autism spectrum disorder (ASD) is associated with abnormal lipid metabolism, such as a high total ratio of omega‐6 to omega‐3 in polyunsaturated fatty acids (PUFAs). PUFAs are metabolized to epoxy fatty acids by cytochrome P450 (CYP); then, dihydroxy fatty acid is produced by soluble epoxide hydrolase. This study examined the association between PUFA metabolites in the cord blood and ASD symptoms and adaptive functioning in children.

**Methods:**

This prospective cohort study utilized cord blood to quantify PUFA metabolites of the CYP pathway. The Autism Diagnostic Observation Schedule (ADOS‐2) and Vineland Adaptive Behaviors Scales, Second Edition (VABS‐II) were used to assess subsequent ASD symptoms and adaptive functioning in children at 6 years. The analysis included 200 children and their mothers.

**Results:**

Arachidonic acid‐derived diols, 11,12‐diHETrE was found to impact ASD symptom severity on the ADOS‐2‐calibrated severity scores and impairment in the socialization domain as assessed by the VABS‐II (*P* = 0.0003; *P* = 0.004, respectively). High levels of 11,12‐diHETrE impact social affect in ASD symptoms (*P* = 0.002), while low levels of 8,9‐diHETrE impact repetitive/restrictive behavior (*P* = 0.003). Notably, there was specificity in the association between diHETrE and ASD symptoms, especially in girls.

**Conclusion:**

These findings suggest that the dynamics of diHETrE during the fetal period is important in the developmental trajectory of children after birth. Given that the role of diol metabolites in neurodevelopment *in vivo* is completely uncharacterized, the results of this study provide important insight into the role of diHETrE and ASD pathophysiology.

Autism spectrum disorder (ASD) is a neurodevelopmental disorder affecting approximately 1 in 44 children by the age of 8.^1^ Additionally, ASD may be linked to immune dysfunction.^2^ Although the exact cause remains elusive, evidence of neuroinflammation is apparent, with postmortem brain studies revealing heightened density of primed microglia^3^ and correlations between specific microglia gene sets and ASD clinical severity.^4^ As the ASD phenotype is recognizable early in life, it is important to focus on its development during pregnancy. Studies on mice have demonstrated that exposure to inflammatory cytokines during pregnancy leads to behavioral impairments reminiscent of ASD.^5^ Similarly, in humans, persistent fever above a certain threshold in pregnancy has been shown to increase the risk of ASD in subsequent children.^6^ Notably, the Norwegian Autism Birth Cohort large study, immune‐related factors (interleukin‐1RA, tumor necrosis factor‐α, serpin E1, vascular cell adhesion molecule 1, vascular endothelial growth factor D, epidermal growth factor, and colony‐stimulating factors 1 and 2) in cord blood plasma and maternal mid‐pregnancy plasma were associated with increased ASD risk.^7^ These findings underscore the significance of maternal immune activation (MIA) as a key ASD risk factor.^8^ Intriguingly, alterations in cytokine and metabolic profiling in cord and maternal mid‐pregnancy plasma from mothers of children with ASD have been found to differ between the sexes.^7^, ^9^

Polyunsaturated fatty acids (PUFAs), represented by arachidonic acid (AA) and metabolites, are key mediators of immune modulatory processes.^10^ The total ratio of omega‐6 to omega‐3 in PUFAs was observed to be elevated in children with ASD.^11^ These PUFAs are regulated by three major enzyme pathways: cyclooxygenase, lipoxygenase, and cytochrome P450 (CYP) pathways. Notably, CYP metabolism forms epoxy fatty acids (EpFAs), which have anti‐inflammatory effects, and dihydroxy fatty acids (diols), which have inflammatory properties. EpFAs act as anti‐inflammatory agents by preventing the transcription of several inflammatory cytokines by decreasing the nuclear translocation of nuclear factor‐kappa B (NF‐κB).^12^ Conversely, diols exert inflammatory effects by driving monocyte motility in response to monocyte chemoattractant protein‐1 (MCP‐1), a monocyte motility protein.^13^ In the context of the central nervous system (CNS), it has been reported that inhibition of soluble epoxide hydrolase (sEH), the enzyme that metabolizes EpFA to diol, selectively induced interleukin (IL)‐10, which has anti‐inflammatory/neuroprotective properties in the microglia.^14^ Furthermore, the AA‐derived EpFA, epoxyeicosatrienoic acid (EET), contributed to neurite outgrowth in neurons.^15^ These findings suggest that CYP‐PUFA metabolites from the CYP pathway may impact fetal development during pregnancy through MIA.^16^

A study also reported that *EPHX2* mRNA expression, the gene encoding sEH, is high in the postmortem brains of patients with ASD (Brodmann areas 9 and 40).^17^ In addition, exposure to high concentrations of glyphosate during pregnancy and lactation induces ASD‐like behavior in the offspring of pregnant mice, along with significant decreases in 8,9‐EET levels and increases in sEH levels in the plasma, prefrontal cortex, hippocampus, and striatum.^18^ The administration of sEH inhibitors to pregnant mice exposed to glyphosate also improved ASD‐like behavior, such as increased grooming time and social interaction deficits in the offspring.^18^ These findings suggest that the presence of CYP‐PUFA metabolites during the fetal period plays an important role in generating a biological background that leads to the development of ASD‐like behavior. Based on this, we hypothesized that the dynamics of CYP‐PUFA metabolites during the fetal period, i.e., lower EpFA levels and/or higher diol levels and/or increased sEH activity, will affect ASD symptoms and/or difficulties with daily functioning in children after birth. To investigate this hypothesis in humans, CYP‐PUFA metabolites will be quantified using cord blood obtained from the birth cohort, and a longitudinal evaluation will be conducted to determine the association with ASD symptoms and ASD adaptive functioning in children after birth.

## Methods

### Study design and participants

**Fig. 1:**
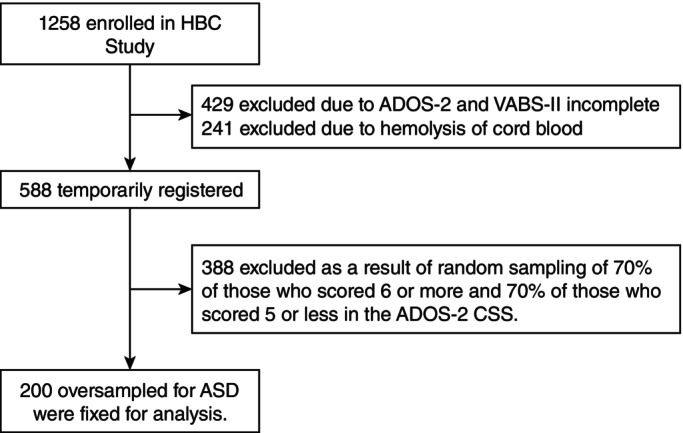
Flow diagram for selecting study population.

This study used a subset of the Hamamatsu Birth Cohort Study for Mothers and Children (HBC Study), an ongoing prospective cohort study of 200 children and their mothers. The HBC Study included women in their first or second trimester of pregnancy who visited Hamamatsu University Hospital or Kato Maternity Clinic between November 2007 and March 2011; their demographic and perinatal profiles are comparable with those of mothers and children in the general Japanese population.^19^, ^20^ Initially, 1258 neonates born from 1138 mothers were enrolled in the HBC Study, whose blood samples were obtained from the clamped umbilical vein immediately after delivery. ASD symptoms and adaptive functioning in these children were assessed at age 6 using the Autism Diagnostic Observation Schedule, Module 3 [Generic or Second Edition (ADOS‐2)] and the Vineland Adaptive Behaviors Scales, Second Edition (VABS‐II). Following procedure for selecting the subset in this study, drawn from the HBC Study, was used (Fig. 1). First, children without complete ADOS‐2 or VABS‐II assessments were excluded. Second, children with hemolyzed serum samples were excluded. These exclusion criteria left 588 children as a base population for the purposeful selection of this study. With an ASD prevalence of approximately 1 in 44,^1^ we intended to include more children showing ASD symptomatology than expected in the HBC Study participants, representative of the general population. Therefore, 70% of those who scored 6 or higher on the ADOS‐2 calibrated severity scores (CSS) (details are discussed below) were randomly selected, and those with CSS scores of 5 or lower were randomly selected, resulting in a final subset of 200 participants. Table S1 compares the main characteristics of the mothers and children included in the HBC Study, which represents the general population, with this study's cohort, wherein ASD was oversampled.

In accordance with the Declaration of Helsinki, the study was approved by the Hamamatsu University Hospital Ethics Committee (No. 20‐82, 21‐114, 22‐29, 24‐67, 24‐237, 25‐143, 25‐283, E14‐062, 17‐037, 17‐037‐3, 20‐233) and the Research Ethics Committee of University of Fukui. All mothers provided written informed consent for themselves and their children to participate in the study.

### Procedures

Immediately after birth, 10–30 mL of blood was collected from the umbilical vein using a vacutainer blood collection system. Samples were held at room temperature for 30 min and then centrifuged at 3500 rpm for 10 min. The resulting supernatant (200 μL), cord blood serum, was divided into aliquots and stored frozen at −80°C until analysis. Liquid chromatography‐mass spectrometry (LC–MS/MS) analysis was performed to quantify CYP‐PUFA metabolites in cord blood serum. Specifically, the lipid fraction containing EpFAs was isolated from 180 μL of serum through solid‐phase extraction with Oasis HLB columns (Waters Corporation, MA, USA). EpFAs were further separated using a high‐performance liquid chromatography system (Nexera LC‐30AD, Shimadzu Corporation, Kyoto, Japan) equipped with an XBridge C18 column (particle size, 3.5 μm; length, 150 mm; inner diameter, 1.0 mm; Waters) and analyzed on a triple quadrupole mass spectrometer (LC–MS‐8040; Shimadzu). Mass spectrometric analysis was conducted in negative‐ion mode, and fatty acid metabolites were identified and quantified by multiple‐reaction monitoring, similar to the determination of other lipid metabolites.^21^ For quantification, calibration curves were prepared for each compound, and recoveries were monitored using the deuterated internal standard (11,12‐EET‐d11, 12,13‐diHOME‐d4, and AA‐d8; Cayman Chemicals, Ann Arbor, Michigan, USA). Data were analyzed using LabSolutions software (Shimadzu). LC–MS/MS analysis was conducted according to Lipidome Lab Co., Ltd. Analytical values below the limit of detection (LOD) were excluded from the analysis. In contrast, analytical values below the limit of quantitation were included in the analysis.

The ADOS‐2 was administered by a research‐reliable administrator (THa) and two other clinically reliable administrators (AO and CN) under the supervision of THa at the age of 6 and was the primary outcome by which ASD symptoms were quantified.^22^, ^23^ The ADOS‐2 is a standardized observational assessment of the presence and severity of social affect (SA) and repetitive/restrictive behavior (RRB). Each item was scored on a scale of 0–3, with higher scores indicating more severe ASD symptoms. The ADOS‐2 diagnostic algorithm derived from raw scores was used to calculate the 10‐level CSS. The CSS was developed within a specific ADOS module group and was less sensitive to age and language than the raw scores.^24^ ADOS‐2 CSS of 1–2 points indicate “negligible to no findings” of ASD symptoms; 3–4 points, a “mild level”; 5–7 points, a “moderate level”; and 8–10 points, a “severe level.”

In addition, the VABS‐II was used to further assess adaptive functioning in participating children at the age of 6.^25^ Adaptive functioning is the ability to perform real‐life skills such as communicating and interacting with others, managing one's health and hygiene, and completing household and chore tasks. The VABS‐II allows the assessment of difficulties in daily living, including ASD‐associated difficulties. A semistructured interview questionnaire was administered to the parents or caregivers of the participants, which assessed four domains: communication (expressive language, receptive language, and writing), daily living skills (community, domestic, and personal), socialization (coping skills, interpersonal, play, and leisure), and motor skills (gross and fine). The raw scores obtained were converted to age‐standardized scores using population norms in Japan. Lower scores indicate severe adaptive functioning impairment.

All routinely recorded birth data, including maternal BMI, age at birth, gestational age, height at birth, birth weight, and perinatal interventions, including cesarean section, were extracted from medical records.

### Statistical analysis

Initially, the correlation between CYP‐PUFA metabolites and ASD symptoms, which were assessed using ADOS‐2, was analyzed by the Spearman rank correlation coefficient. Then, multivariate linear regression analysis with covariates was performed to confirm whether CYP‐PUFA metabolites affected ASD symptoms after adjusting for confounding factors. Since the objective variable ADOS‐2 CSS showed non‐normality (Kolmogorov–Smirnov test), log10 transformation was performed to approximate normality. Covariates were selected based on previous studies^26^, ^27^ and included late‐pregnancy BMI, maternal age, gestational age, birth weight, the child's sex, parity, and delivery method (cesarean section). The ADOS‐2 subdomains SA and RRB were similarly subjected to multivariate linear regression analysis. The correlation of CYP‐PUFA metabolites with ASD adaptive functioning, as indicated by the VABS‐II, was also examined by the Spearman rank correlation coefficient. The statistical significance level was set at: *P*‐values of 0.01 to account for multiplicity issues. Asterisks indicate *P*‐values (**P < 0.001, *P < 0.01). All analyses were performed using IBM SPSS Statistics version 28 (IBM Corp., Armonk, NY, USA).

## Results

A total of 200 participants who completed the ASD symptom and adaptive functioning assessments were included in the analysis. In this cohort study, the mean maternal age was 32.3 years (standard deviation SD = 5.0), 106 children were boys, and 94 were girls. The severity of ASD based on the ADOS‐2 CSS in the 200 participants was “negligible to no findings” in 98 children (49%), “mild” in 29 (14.5%), “moderate” in 39 (19.5%), and “severe” in 34 (17%). Other participant characteristics are shown in Table 1.

**Table 1:** Characteristics of participating children and their parents.

	Sex‐inclusive	Boys	Girls
Participant chacteristics	*n* = 200	*n* = 106	*n* = 94
Maternal characteristics			
Age (years)	32.3 (5.0)	32.2 (5.0)	32.4 (5.0)
BMI in late pregnancy	25.4 (3.7)	26.1 (3.9)	24.7 (3.3)
Delivery type			
Vaginal	157 (78.5%)	87 (82.1%)	70 (74.5%)
Cesarean	43 (21.5%)	19 (17.9%)	24 (25.5%)
Gestational age (weeks)	39.1 (1.2)	39.1 (1.2)	39.1 (1.3)
Parity			
Primipara	100 (50%)	52 (49.1%)	48 (51.1%)
Multipara	100 (50%)	54 (50.9%)	46 (48.9%)
Children's characteristics at birth			
Birth weight (g)	2995.4 (394.8)	3054.6 (433.8)	2928.7 (335.6)
Children's characteristics at age 6			
ADOS‐2			
Total score	6.2 (5.7)	6.7 (6.3)	5.7 (5.0)
CSS	3.8 (3.0)	4.0 (3.3)	3.5 (2.7)
Severity according to the ADOS‐2 CSS			
CSS of 1–2	98 (49%)	52 (49.1%)	46 (48.9%)
CSS of 3–4	29 (14.5%)	12 (11.3%)	17 (18.1%)
CSS of 5–7	39 (19.5%)	21 (19.8%)	18 (19.2%)
CSS of 8–10	34 (17%)	21 (19.8%)	13 (13.8%)

Date are expressed as mean (Standard Deviation: SD) or *N* (%).

ADOS‐2, Autism Diagnostic Observation Schedule; BMI, Body Mass Index; CSS, Calibrated Severity Score.

### Relationship between CYP pathway PUFA metabolite profiles in cord blood with ASD symptoms and adaptive functions

CYP‐PUFA metabolites are divided into LA‐derived, AA‐derived, eicosapentaenoic acid (EPA)‐derived, and docosahexaenoic acid (DHA)‐derived metabolites. In cord blood serum, including both sexes, the average total concentrations of LA‐derived epoxy octadecenoic acid (EpOME) and diHOME were 1075.2 pg./mL (SD = 720.1) and 1303.9 pg./mL (SD = 464.4), respectively. The average total concentrations of epoxy eicosatrienoic acid (EET) and diHETrE were 518.3 pg./mL (SD = 464.5) and 3345.7 pg./mL (SD = 955.2), respectively. EPA‐derived eicosatetraenoic acid and dihydroxy eicosatetraenoic acid (diHETE) were 233.9 pg./mL (SD = 27.0) and 6275.5 pg./mL (SD = 3103.9), respectively. In contrast, DHA‐derived epoxy docosapentaenoic acid and dihydroxy docosapentaenoic acid were 439.1 pg./mL (SD = 661.1) and 5718.6 pg./mL (SD = 1895.1), respectively (Table 2).

**Table 2:** Correlation of ASD characteristics with PUFA metabolites of the CYP pathway in cord blood profile.

			ADOS‐2 CSS												
	Sex‐inclusive					Boys					Girls				
Metabolites	*n*	Mean (SD)	r	95% CI	*P*	*n*	Mean (SD)	r	95% CI	*P*	*n*	Mean (SD)	r	95% CI	*P*
Linoleic acid‐derived															
9,10‐EpOME	199	472.0 (393.6)	−0.022	−0.165 to 0.121	0.755	105	470.6 (370.8)	0.055	−0.144 to 0.249	0.577	94	473.5 (419.7)	−0.121	−0.321 to 0.090	0.245
12,13‐EpOME	199	608.7 (339.0)	−0.028	−0.170 to 0.116	0.695	105	626.6 (335.9)	0.008	−0.190 to 0.205	0.936	94	588.6 (343.0)	−0.073	−0.277 to 0.138	0.484
Total EpOME	200	1075.2 (720.1)	−0.035	−0.177 to 0.108	0.622	106	1086.8 (697.1)	0.016	−0.181 to 0.211	0.873	94	1062.1 (748.8)	−0.108	−0.310 to 0.103	0.299
9,10‐diHOME	200	44.3 (24.8)	0.097	−0.046 to 0.237	0.171	106	45.8 (28.8)	0.131	−0.067 to 0.319	0.181	94	42.5 (19.4)	0.051	−0.160 to 0.256	0.628
12,13‐diHOME	200	1259.7 (441.5)	0.080	−0.064 to 0.220	0.263	106	1309.6 (540.9)	0.142	−0.056 to 0.329	0.147	94	1203.4 (284.1)	−0.015	−0.222 to 0.194	0.888
Total diHOME	200	1303.9 (464.4)	0.080	−0.063 to 0.221	0.258	106	1355.4 (568.2)	0.138	−0.060 to 0.325	0.158	94	1245.8 (301.0)	−0.010	−0.218 to 0.199	0.926
9,10‐diHOME/9,10‐EpOME	199	0.1 (0.087)	0.106	−0.037 to 0.246	0.135	105	0.1 (0.09)	0.040	−0.159 to 0.235	0.687	94	0.1 (0.1)	0.197	−0.012 to 0.389	0.057
12,13‐diHOME/12,13‐EpOME	199	2.3 (0.925)	0.122	−0.210 to 0.261	0.085	105	2.4 (1.1)	0.147	−0.052 to 0.334	0.135	94	2.3 (0.8)	0.073	−0.138 to 0.277	0.487
Total diHOME/Total EpOME	200	1.5 (0.639)	0.112	−0.031 to 0.251	0.113	106	1.5 (0.7)	0.103	−0.095 to 0.294	0.292	94	1.4 (0.5)	0.131	−0.080 to 0.330	0.210
Arachidonic acid‐derived														
5,6‐EET	99	217.3 (172.4)	−0.107	−0.303 to 0.099	0.293	51	232.0 (158.9)	−0.187	−0.447 to 0.102	0.188	48	201.7 (186.1)	−0.062	−0.348 to 0.234	0.676
8,9‐EET	94	305.3 (210.3)	0.004	−0.205 to 0.212	0.970	42	313.5 (209.8)	−0.056	−0.362 to 0.261	0.727	52	298.7 (212.5)	0.030	−0.253 to 0.308	0.834
11,12‐EET	128	119.1 (77.6)	−0.002	−0.180 to 0.177	0.983	60	121.8 (64.5)	−0.152	−0.398 to 0.113	0.246	68	116.6 (87.9)	0.126	−0.123 to 0.360	0.307
14,15‐EET	67	160.3 (101.3)	0.047	−0.202 to 0.291	0.705	31	164.2 (83.5)	−0.176	−0.507 to 0.201	0.344	36	156.9 (115.5)	0.252	−0.094 to 0.543	0.139
Total EET	147	518.3 (464.5)	0.036	−0.132 to 0.201	0.668	69	542.0 (442.5)	0.083	−0.164 to 0.320	0.498	78	477.3 (485.0)	−0.045	−0.271 to 0.186	0.695
5,6‐diHETrE	118	224.8 (123.1)	0.089	−0.098 to 0.271	0.336	53	245.1 (149.1)	−0.095	−0.363 to 0.188	0.498	65	208.2 (94.9)	0.199	−0.055 to 0.428	0.113
8,9‐diHETrE	199	1116.7 (419.6)	0.069	−0.075 to 0.210	0.330	106	1159.1 (430.1)	−0.157	−0.342 to 0.041	0.109	93	1068.3 (404.1)	0.337	0.137 to 0.510	0.0009**
11,12‐diHETrE	200	836.8 (289.3)	0.196	0.054 to 0.329	0.006*	106	858.5 (299.0)	0.158	−0.039 to 0.344	0.105	94	812.4 (277.4)	0.229	0.022 to 0.418	0.026
14,15‐diHETrE	200	1365.2 (394.8)	0.221	0.081 to 0.353	0.002*	106	1392.4 (407.6)	0.270	0.078 to 0.443	0.005*	94	1334.5 (379.7)	0.137	−0.074 to 0.335	0.189
Total diHETrE	200	3445.7 (955.2)	0.192	0.050 to 0.325	0.007*	106	3532.5 (972.9)	0.110	−0.088 to 0.300	0.261	94	3347.9 (930.3)	0.282	0.078 to 0.463	0.006*
5,6‐diHETrE/5,6‐EET	76	1.4 (0.963)	0.168	−0.067 to 0.385	0.147	34	1.5 (1.0)	0.195	−0.163 to 0.508	0.269	42	1.3 (1.0)	0.138	−0.182 to 0.432	0.383
8,9‐diHETrE/8,9‐EET	94	4.4 (2.534)	0.080	−0.131 to 0.283	0.446	42	4.4 (2.6)	0.042	−0.274 to 0.350	0.791	52	4.3 (2.5)	0.152	−0.134 to 0.415	0.282
11,12‐diHETrE/11,12‐EET	128	8.8 (5.269)	0.157	−0.022 to 0.327	0.076	60	8.5 (5.1)	0.236	−0.026 to 0.469	0.069	68	9.0 (5.5)	0.100	−0.149 to 0.337	0.418
14,15‐diHETrE/14,15‐EET	67	10.8 (6.480)	0.003	−0.244 to 0.250	0.980	31	10.9 (7.5)	0.172	−0.205 to 0.504	0.355	36	10.8 (5.6)	−0.118	−0.438 to 0.229	0.493
Total diHETrE/Total EET	147	12.6 (12.559)	0.013	−0.153 to 0.180	0.871	69	12.6 (12.7)	−0.073	−0.311 to 0.174	0.552	78	12.7 (12.6)	0.135	−0.097 to 0.353	0.240
Eicosapentaenoic acid‐derived															
5,6‐EpETE	0	‐	‐	‐	‐	0	‐	‐	‐	‐	0	‐	‐	‐	‐
8,9‐EpETE	2	220.7 (20.3)	1.000	‐	‐	1	235.1 (‐)	‐	‐	‐	1	206.3 (‐)	‐	‐	‐
11,12‐EpETE	0	‐	‐	‐	‐	0	‐	‐	‐	‐	0	‐	‐	‐	‐
14,15‐EpETE	0	‐	‐	‐	‐	0	‐	‐	‐	‐	0	‐	‐	‐	‐
17,18‐EpETE	1	260.2 (‐)	‐	‐	‐	0	‐	‐	‐	‐	1	260.2 (‐)	‐	‐	‐
Total EpETE	3	233.9 (27.0)	0.500	‐	0.667	1	235.1 (‐)	‐	‐	‐	2	233.3 (38.1)	‐	‐	‐
5,6‐diHETE	0	‐	‐	‐	‐	0	‐	‐	‐	‐	0	‐	‐	‐	‐
8,9‐diHETE	29	334.6 (138.3)	0.314	−0.071 to 0.617	0.097	14	324.1 (94.4)	0.322	−0.268 to 0.736	0.262	15	344.4 (172.5)	0.457	−0.088 to 0.792	0.087
11,12‐diHETE	159	125.2 (49.9)	−0.018	−0.178 to 0.142	0.821	81	132.2 (55.3)	−0.042	−0.264 to 0.184	0.708	78	118.0 (42.7)	−0.023	−0.251 to 0.207	0.842
14,15‐diHETE	159	701.9 (292.3)	0.060	−0.101 to 0.218	0.455	76	740.4 (260.4)	0.008	−0.224 to 0.240	0.942	83	666.7 (316.2)	0.037	−0.187 to 0.256	0.742
17,18‐diHETE	199	5597.4 (2810.5)	0.111	−0.032 to 0.251	0.117	106	5631.8 (3135.1)	0.158	−0.039 to 0.344	0.106	93	5558.1 (2403.9)	0.053	−0.158 to 0.260	0.614
Total diHETE	200	6275.5 (3103.9)	0.116	−0.027 to 0.254	0.102	106	6306.4 (3399.0)	0.196	−0.001 to 0.377	0.044	94	6240.5 (2751.2)	0.012	−0.197 to 0.220	0.906
5,6‐diHETE/5,6‐EpETE	0	‐	‐	‐	‐	0	‐	‐	‐	‐	0	‐	‐	‐	‐
8,9‐diHETE/8,9‐EpETE	0	‐	1	‐	‐	0	‐	‐	‐	‐	0	‐	‐	‐	‐
11,12‐diHETE/11,12‐EpETE	0	‐	‐	‐	‐	0	‐	‐	‐	‐	0	‐	‐	‐	‐
14,15‐diHETE/14,15‐EpETE	0	‐	‐	‐	‐	0	‐	‐	‐	‐	0	‐	‐	‐	‐
17,18‐diHETE/17,18‐EpETE	1	15.2 (‐)	‐	‐	‐	0	‐	‐	‐	‐	1	15.2 (‐)	‐	‐	‐
Total diHETE/Total EpETE	3	19.2 (2.0)	0.5	‐	0.667	1	20.6 (‐)	‐	‐	‐	2	18.5 (2.2)	‐	‐	‐
Docosahexaenoic acid‐derived															
4,5‐EpDPA	0	‐	‐	‐	‐	0	‐	‐	‐	‐	0	‐	‐	‐	‐
7,8‐EpDPA	15	317.0 (125.6)	−0.066	−0.570 to 0.475	0.816	8	358.8 (124.4)	−0.169	−0.790 to 0.624	0.690	7	269.1 (117.4)	0.037	−0.750 to 0.780	0.937
10,11‐EpDPA	144	137.0 (94.6)	−0.130	−0.292 to 0.039	0.119	68	138.5 (96.3)	−0.142	−0.374 to 0.107	0.247	76	135.7 (93.8)	−0.137	−0.358 to 0.098	0.238
13,14‐EpDPA	7	410.6 (185.7)	0.694	−0.151 to 0.953	0.083	5	510.6 (83.0)	0.224	−0.833 to 0.929	0.718	2	160.6 (66.8)	‐	‐	‐
16,17‐EpDPA	35	200.9 (193.5)	0.103	−0.248 to 0.430	0.557	17	240.4 (245.6)	−0.013	−0.502 to 0.483	0.961	18	163.6 (122.7)	0.039	−0.448 to 0.508	0.879
19,20‐EpDPA	46	703.3 (703.6)	−0.125	−0.408 to 0.180	0.408	28	834.6 (852.3)	−0.271	−0.592 to 0.125	0.163	18	498.9 (289.8)	0.135	−0.367 to 0.576	0.593
Total EpDPA	152	439.1 (661.1)	−0.134	−0.292 to 0.030	0.099	75	536.9 (834.6)	−0.148	−0.369 to 0.088	0.205	77	317.4 (399.7)	−0.146	−0.364 to 0.087	0.204
4,5‐diHDoPE	0	‐	‐	‐	‐	‐	‐	‐	‐	‐	‐	‐	‐	‐	‐
7,8‐diHDoPE	53	417.0 (263.9)	−0.295	−0.530 to −0.019	0.032	26	444.7 (350.0)	−0.374	−0.672 to 0.027	0.060	27	390.3 (141.5)	−0.219	−0.561 to 0.187	0.272
10,11‐diHDoPE	197	255.4 (112.4)	0.052	−0.093 to 0.194	0.468	104	254.5 (106.5)	0.102	−0.098 to 0.294	0.302	93	256.4 (119.2)	0.004	−0.206 to 0.213	0.970
13,14‐diHDoPE	200	893.9 (311.4)	0.129	−0.014 to 0.266	0.070	106	903.1 (316.0)	0.142	−0.055 to 0.329	0.146	94	883.5 (307.4)	0.121	−0.090 to 0.321	0.245
16,17‐diHDoPE	196	646.9 (193.3)	0.179	0.036 to 0.315	0.012	103	657.2 (185.4)	0.248	0.052 to 0.426	0.011	93	635.3 (202.1)	0.077	−0.134 to 0.282	0.462
19,20‐diHDoPE	200	3828.8 (1313.9)	0.126	−0.017 to 0.264	0.076	106	3893.6 (1257.5)	0.230	0.035 to 0.407	0.018	94	3755.7 (1377.8)	−0.029	−0.236 to 0.181	0.785
Total diHDoPE	200	5718.6 (1895.1)	0.137	−0.006 to 0.274	0.054	106	5794.0 (1818.0)	0.227	0.032 to 0.405	0.020	94	5633.6 (1984.8)	−0.003	−0.211 to 0.206	0.981
4,5‐diHDoPE/4,5‐EpDPA	0	‐	‐	‐	‐	‐	‐	‐	‐	‐	‐	‐	‐	‐	‐
7,8‐diHDoPE/7,8‐EpDPA	6	1.5 (0.4)	−0.265	−0.893 to 0.713	0.612	3	1.5 (0.5)	−0.866	‐	0.333	3	1.5 (0.3)	0.500	‐	0.667
10,11‐diHDoPE/10,11‐EpDPA	142	2.4 (1.3)	0.105	−0.066 to 0.270	0.213	67	2.4 (1.2)	0.148	−0.103 to 0.381	0.232	75	2.4 (1.3)	0.078	−0.158 to 0.306	0.504
13,14‐diHDoPE/13,14‐EpDPA	7	2.7 (1.6)	−0.386	−0.889 to 0.539	0.393	5	1.9 (0.5)	0.224	−0.833 to 0.929	0.718	2	4.9 (1.3)	‐	‐	‐
16,17‐diHDoPE/16,17‐EpDPA	35	0.002 (0.0005)	0.058	−0.290 to 0.393	0.740	17	4.6 (3.1)	−0.166	−0.609 to 0.356	0.525	18	4.8 (2.3)	−0.405	−0.740 to 0.092	0.096
19,20‐diHDoPE/19,20‐EpDPA	46	8.9 (5.9)	0.181	−0.125 to 0.454	0.230	28	8.0 (6.4)	0.206	−0.192 to 0.546	0.293	18	10.2 (4.9)	0.134	−0.368 to 0.576	0.596
Total diHDoPE/Total EpDPA	152	41.3 (36.5)	0.129	−0.035 to 0.287	0.113	75	40.5 (41.0)	0.188	−0.048 to 0.404	0.107	77	42.2 (31.8)	0.082	−0.151 to 0.307	0.478

Asterisk indicate *P < 0.01, spearman rank correlation coefficient. 95% CI, 95% confidence interval; ADOS‐2 CSS, Autism Diagnostic Observation Schedule calibrated severity scores; diHDoPE, dihydroxy docosapentaenoic acid; diHETE, dihydroxy eicosatetraenoic acid; diHETrE, dihydroxy eicosatrienoic acid; diHOME, dihydroxy octadecenoic acid; EET, epoxy eicosatrienoic acid; EpDPA, epoxy docosapentaenoic acid; EpETE, epoxy eicosatetraenoic acid; EpOME, epoxy octadecenoic acid; SD, standard deviation.

Although we have previously reported that PUFAs are associated with ASD,^28^ the role of downstream metabolites of PUFAs remains unclear. First, we analyzed the correlation between these CYP‐PUFA metabolites and ADOS‐2, which is used to assess ASD symptoms and is widely considered the gold standard in assessing ASD (Table 2). We found a significant correlation between the ADOS‐2 CSS and AA‐derived diol, and no other significant correlations were found. Specifically, in participants sex‐inclusive, the higher the 11,12‐diHETrE (*r* = 0.196, 95% confidence interval [CI] = 0.054 to 0.329, *P* = 0.006), 14,15‐diHETrE (*r* = 0.221, 95% CI = 0.081 to 0.353, *P* = 0.002), or total diHETrE (*r* = 0.192, 95% CI = 0.050 to 0.325, *P* = 0.007), the higher the ADOS‐2 CSS. In other words, the higher the cord blood concentration of these diHETrEs, the more severe the ASD symptoms exhibited by the patient. The higher the 14,15‐diHETrE levels in boys (*r* = 0.270, 95% CI = 0.078–0.443, *P* = 0.005) and the higher the 8,9‐diHETrE levels (*r* = 0.337, 95% CI = 0.137–0.510, *P* = 0.0009) or total diHETrE levels in girls (*r* = 0.282, 95% CI = 0.078–0.463, *P* = 0.006), the higher the ADOS‐2 CSS.

Therefore, we examined the relationship between AA‐derived metabolites and ASD symptoms in more detail using multivariate linear regression analysis, controlling for the influence of confounding factors (Table 3). Consistent with the results of the correlation analysis, no statistical significance was found for EET. By contrast, 11,12‐diHETrE, 14,15‐diHETrE, and total diHETrE were significantly related to ADOS‐2 CSS (*β* = 0.251, 95% CI = 0.0004–0.001, *P* = 0.0003; *β* = 0.191, 95% CI = 0.0001–0.001, *P* = 0.007; *β* = 0.227, 95% CI = 0.00008–0.0003, *P* = 0.001; respectively). For example, an increase of 1.0 in the level of total diHETrE indicates that the predicted value of the log‐transformed ADOS‐2 CSS score is 0.227 higher. By further exponentiating the regression coefficients, we found that an increase of 1.0 in total diHETrE levels results in a 1.26‐fold increase in the predicted value of the ADOS‐2 CSS score. Based on sex, no association was found between AA‐derived metabolites and ASD symptoms in boys, whereas in girls, 8,9‐diHETrE, 11,12‐diHETrE, and total diHETrE levels were significantly associated (*β* = 0.395, 95% CI = 0.001–0.004, *P* = 0.0001; *β* = 0.267, 95% CI = 0.001–0.005, *P* = 0.009; *β* = 0.317, 95% CI = 0.0004–0.002, *P* = 0.002; respectively). Further, the relationship between AA‐derived metabolites and SA and RRB was investigated (Table 4). A significant positive association was found for SA at 11,12‐diHETrE, and total diHETrE (*β* = 0.222, 95% CI = 0.0003–0.001, *P* = 0.002; *β* = 0.206, 95% CI = 0.00007–0.0004, *P* = 0.003). In contrast, a negative association was observed for RRB at 8,9‐diHETrE (*β* = −0.247, 95% CI = −0.001 to −0.0002, *P* = 0.0003). Significant association with diHETrE only in SA was seen in girls (*β* = 0.357, 95% CI = 0.0004–0.001, *P* = 0.0007; *β* = 0.275, 95% CI = 0.0003–0.002, *P* = 0.008; *β* = 0.299, 95% CI = 0.0001–0.001, *P* = 0.004), while the association was only found in RRB in boys (*β* = −0.278, 95% CI = −0.001 to −0.0002, *P* = 0.0003).

**Table 3:** Association of AA metabolites with ASD after adjustment for confounding factors.

	ADOS‐2 CSS								
	Sex‐inclusive			Boys			Girls		
Metabolites	β	95% CI	*P*	β	95% CI	*P*	β	95% CI	*P*
AA‐derived									
5,6‐EET	−0.114	−0.002 to 0.0004	0.259	−0.136	−0.009 to 0.003	0.347	−0.159	−0.006 to 0.002	0.332
8,9‐EET	0.05	−0.001 to 0.001	0.639	−0.015	−0.006 to 0.005	0.929	0.135	−0.002 to 0.005	0.373
11,12‐EET	−0.026	−0.002 to 0.002	0.769	−0.063	−0.017 to 0.011	0.654	0.069	−0.006 to 0.010	0.603
14,15‐EET	−0.079	−0.003 to 0.001	0.535	−0.093	−0.018 to 0.011	0.616	0.074	−0.007 to 0.010	0.676
Total EET	0.011	−0.0003 to 0.0003	0.899	0.045	−0.001 to 0.002	0.717	0.001	−0.001 to 0.001	0.992
5,6‐diHETrE	0.119	−0.0005 to 0.002	0.206	−0.049	−0.008 to 0.005	0.747	0.283	0.001 to 0.014	0.026
8,9‐diHETrE	0.142	0.000004 to 0.001	0.047	−0.070	−0.002 to 0.001	0.465	0.395	0.001 to 0.004	0.0001**
11,12‐diHETrE	0.251	0.0004 to 0.001	0.0003**	0.227	0.0004 to 0.005	0.020	0.267	0.001 to 0.005	0.009*
14,15‐diHETrE	0.191	0.0001 to 0.001	0.007*	0.202	0.00008 to 0.003	0.039	0.147	−0.0004 to 0.003	0.162
Total diHETrE	0.227	0.00008 to 0.0003	0.001*	0.121	−0.0002 to 0.001	0.210	0.317	0.0004 to 0.002	0.002*
5,6‐diHETrE/5,6‐EET	0.110	−0.105 to 0.300	0.340	0.123	−0.884 to 1.700	0.522	0.048	−0.687 to 0.902	0.785
8,9‐diHETrE/8,9‐EET	0.012	−0.066 to 0.074	0.908	−0.067	−0.538 to 0.371	0.712	0.030	−0.253 to 0.313	0.831
11,12‐diHETrE/11,12‐EET	0.163	−0.002 to 0.055	0.068	0.220	−0.035 to 0.317	0.115	0.035	−0.102 to 0.135	0.780
14,15‐diHETrE/14,15‐EET	−0.064	−0.041 to 0.024	0.604	−0.106	−0.216 to 0.128	0.601	−0.268	−0.277 to 0.015	0.076
Total diHETrE/Total EET	−0.017	−0.013 to 0.010	0.839	−0.165	−0.106 to 0.021	0.183	0.055	−0.037 to 0.060	0.644

Adjustment for confounders included the following: late‐pregnancy BMI, maternal age at birth, gestational age at birth, birth weight, sex of the child, parity, and delivery method (cesarean section). Asterisks indicate **P < 0.001, *P < 0.01, multivariate linear regression analysis. 95% CI, 95% confidence interval; AA, arachidonic acid; ADOS‐2 CSS, Autism Diagnostic Observation Schedule calibrated severity scores; diHETrE, dihydroxy eicosatrienoic acid; EET, epoxy eicosatrienoic acid.

**Table 4:** Association of AA metabolites with social affect and repetitive/restrictive behavior.

	Sex‐inclusive						Boys						Girls					
	SA			RRB			SA			RRB			SA			RRB		
Metabolites	β	95% CI	*P*	β	95% CI	*P*	β	95% CI	*P*	β	95% CI	*P*	β	95% CI	*P*	β	95% CI	*P*
AA‐derived																		
5,6‐EET	−0.060	−0.001 to 0.001	0.554	−0.233	−0.002 to −0.0002	0.013	−0.099	−0.002 to 0.001	0.503	−0.229	−0.002 to 0.0002	0.111	0.038	−0.001 to 0.002	0.818	−0.287	−0.002 to 0.00004	0.060
8,9‐EET	−0.015	−0.001 to 0.001	0.888	−0.159	−0.001 to 0.0001	0.116	0.049	−0.001 to 0.002	0.780	−0.253	−0.005 to 0.0001	0.124	0.123	−0.001 to 0.002	0.444	0.065	−0.001 to 0.001	0.629
11,12‐EET	0.010	−0.002 to 0.002	0.914	−0.165	−0.003 to 0.00004	0.056	0.012	−0.004 to 0.004	0.931	−0.233	−0.005 to 0.0003	0.085	0.128	−0.001 to 0.004	0.328	−0.188	−0.003 to 0.0003	0.125
14,15‐EET	0.045	−0.002 to 0.003	0.728	−0.306	−0.004 to −0.0004	0.014	−0.082	−0.006 to 0.004	0.692	−0.377	−0.006 to −0.0005	0.023	0.178	−0.002 to 0.005	0.316	−0.167	−0.003 to 0.001	0.387
Total EET	0.015	−0.0003 to 0.0004	0.860	−0.133	−0.0004 to 0.00004	0.104	0.074	−0.0004 to 0.001	0.522	−0.090	−0.0005 to 0.0002	0.458	0.093	−0.0003 to 0.001	0.467	−0.184	−0.0005 to 0.00005	0.107
5,6‐diHETrE	0.112	−0.001 to 0.002	0.234	−0.194	−0.002 to −0.0001	0.031	0.044	−0.002 to 0.002	0.774	−0.264	−0.002 to 0.00009	0.067	0.263	0.00008 to 0.005	0.044	−0.154	−0.002 to 0.0005	0.199
8,9‐diHETrE	0.168	0.00007 to 0.001	0.018	−0.247	−0.001 to −0.0002	0.0003**	0.007	−0.0004 to 0.0005	0.940	−0.278	−0.001 to −0.0002	0.003*	0.357	0.0004 to 0.001	0.0007**	−0.185	−0.001 to 0.00003	0.078
11,12‐diHETrE	0.222	0.0003 to 0.001	0.002*	−0.046	−0.0004 to 0.0002	0.509	0.206	0.00004 to 0.001	0.037	−0.020	−0.0005 to 0.0004	0.843	0.275	0.0003 to 0.002	0.008*	−0.001	−0.0004 to 0.0004	0.992
14,15‐diHETrE	0.146	0.00002 to 0.001	0.039	−0.007	−0.0002 to 0.0002	0.915	0.186	−0.00002 to 0.001	0.061	0.068	−0.0002 to 0.0004	0.492	0.151	−0.0002 to 0.001	0.158	−0.043	−0.0004 to 0.0002	0.679
Total diHETrE	0.206	0.00007 to 0.0004	0.003*	−0.133	−0.0002 to 0.000001	0.053	0.155	−0.00004 to 0.0004	0.114	−0.111	−0.0002 to 0.00006	0.256	0.299	0.0001 to 0.001	0.004*	−0.090	−0.0002 to 0.00007	0.384
5,6‐diHETrE / 5,6‐EET	0.112	−0.110 to 0.328	0.325	0.073	−0.097 to 0.198	0.497	0.177	−0.196 to 0.524	0.357	−0.089	−0.312 to 0.193	0.633	−0.003	−0.336 to 0.331	0.988	0.062	−0.151 to 0.222	0.705
8,9‐diHETrE/8,9‐EET	0.074	−0.051 to 0.107	0.485	−0.033	−0.062 to 0.044	0.739	−0.191	−0.206 to 0.066	0.304	0.016	−0.090 to 0.098	0.930	0.160	−0.050 to 0.173	0.274	−0.179	−0.090 to 0.013	0.141
11,12‐diHETrE/11,12‐EET	0.117	−0.010 to 0.052	0.187	0.154	−0.002 to 0.040	0.072	0.170	−0.021 to 0.086	0.233	0.218	−0.007 to 0.063	0.109	0.079	−0.029 to 0.055	0.528	0.086	−0.014 to 0.031	0.463
14,15‐diHETrE/14,15‐EET	−0.146	−0.058 to 0.015	0.240	0.224	−0.002 to 0.049	0.066	−0.137	−0.075 to 0.040	0.541	0.182	−0.018 to 0.052	0.337	−0.250	−0.098 to 0.009	0.101	0.051	−0.028 to 0.038	0.764
Total diHETrE/Total EET	0.002	−0.012 to 0.013	0.977	−0.062	−0.011 to 0.005	0.441	−0.119	−0.029 to 0.010	0.343	−0.194	−0.023 to 0.002	0.110	0.068	−0.013 to 0.023	0.572	0.051	−0.007 to 0.011	0.637

Adjustment for confounders included the following: late‐pregnancy BMI, maternal age at birth, gestational age at birth, birth weight, sex of the child, parity, and delivery method (cesarean section). Asterisks indicate **P < 0.001, *P < 0.01, multivariate linear regression analysis. 95% CI, 95% confidence interval; AA, arachidonic acid; diHETrE, dihydroxy eicosatrienoic acid; EET, epoxy eicosatrienoic acid; RRB, repetitive/restrictive behavior; SA, social affect.

Next, we focused on AA‐derived metabolites associated with ADOS‐2 CSS scores and examined their correlation with ASD adaptive functions using VABS‐II. Communication, daily living skills, socialization, and motor domains were not significantly correlated with adaptive functions. In contrast, a significant negative correlation was found with 11,12‐diHETrE in coping skills, a subdomain of the socialization domain (*r* = −0.202, 95% CI = −0.335 to −0.061, *P* = 0.004) (Table 5). In other words, lower degrees of coping skills regarding ASD symptoms were associated with higher levels of 11,12‐diHETrE. In addition, we confirmed a statistically significant negative correlation trend for 11,12‐diHETrE in the socialization domain of Interpersonal (*r* = −0.165, 95% CI = −0.301 to −0.023, *P* = 0.019). A significant difference was found in girls only in 5,6‐EET and in the receptive language of the communication domain when examined separately based on sex (*r* = 0.411, 95% CI = 0.136–0.628, *P* = 0.004) (Table 5).

**Table 5:** Correlation of VABS‐II with arachidonic acid‐derived metabolites.

	5,6‐EET			8,9‐EET			11,12‐EET			14,15‐EET			Total EET		
		*n* = 99			*n* = 94			*n* = 128			*n* = 67			*n* = 147	
VABS‐Ⅱ	*r*	95% CI	*P*	*r*	95% CI	*P*	*r*	95% CI	*P*	*r*	95% CI	*P*	*r*	95% CI	*P*
Communication	0.058	−0.147 to 0.258	0.567	0.049	−0.161 to 0.255	0.641	−0.064	−0.240 to 0.116	0.471	0.068	−0.182 to 0.310	0.585	0.030	−0.137 to 0.196	0.716
Expressive Laguage	0.045	−0.159 to 0.246	0.656	0.053	−0.158 to 0.258	0.615	−0.022	−0.199 to 0.158	0.808	0.043	−0.207 to 0.287	0.733	0.060	−0.107 to 0.225	0.468
Receptive Language	0.173	−0.031 to 0.364	0.086	0.063	−0.147 to 0.268	0.544	−0.044	−0.221 to 0.135	0.619	0.168	−0.082 to 0.399	0.173	0.115	−0.052 to 0.277	0.164
Writing	−0.073	−0.272 to 0.132	0.473	0.033	−0.176 to 0.240	0.749	−0.077	−0.252 to 0.103	0.386	−0.035	−0.280 to 0.214	0.779	−0.101	−0.263 to 0.066	0.222
Daily Living Skills	0.053	−0.152 to 0.253	0.605	0.071	−0.140 to 0.275	0.497	−0.023	−0.201 to 0.156	0.794	0.052	−0.198 to 0.295	0.679	0.015	−0.152 to 0.181	0.855
Community	0.056	−0.148 to 0.256	0.580	0.129	−0.082 to 0.328	0.216	−0.056	−0.232 to 0.124	0.528	−0.008	−0.255 to 0.239	0.948	0.029	−0.139 to 0.194	0.730
Domestic	0.025	−0.179 to 0.227	0.805	0.110	−0.101 to 0.311	0.293	−0.038	−0.215 to 0.141	0.67	−0.045	−0.289 to 0.204	0.717	0.068	−0.099 to 0.232	0.410
Personal	0.023	−0.181 to 0.225	0.821	−0.004	−0.212 to 0.204	0.968	−0.010	−0.188 to 0.169	0.913	0.051	−0.198 to 0.295	0.680	0.005	−0.161 to 0.172	0.948
Socialization	0.059	−0.146 to 0.259	0.561	0.094	−0.117 to 0.297	0.367	−0.016	−0.194 to 0.163	0.862	0.032	−0.217 to 0.277	0.797	0.168	−0.217 to 0.277	0.042
Coping Skills	0.115	−0.090 to 0.311	0.258	0.110	−0.101 to 0.311	0.292	0.023	−0.156 to 0.201	0.795	0.131	−0.120 to 0.366	0.291	0.194	0.029 to 0.350	0.018
Interpersonal	0.156	−0.049 to 0.348	0.123	0.078	−0.133 to 0.282	0.455	0.049	−0.130 to 0.226	0.581	−0.055	−0.298 to 0.195	0.66	0.166	−0.001 to 0.323	0.045
Play and Leisure	−0.054	−0.254 to 0.151	0.595	0.045	−0.165 to 0.251	0.670	−0.091	−0.266 to 0.089	0.305	0.007	−0.240 to 0.254	0.955	0.088	−0.080 to 0.251	0.290
Motor	−0.0003	−0.203 to 0.203	0.997	0.047	−0.163 to 0.253	0.651	−0.017	−0.195 to 0.162	0.845	−0.001	−0.248 to 0.246	0.992	−0.003	−0.170 to 0.164	0.970
Gross	−0.056	−0.257 to 0.148	0.579	0.035	−0.175 to 0.242	0.738	−0.086	−0.261 to 0.094	0.332	−0.008	−0.254 to 0.240	0.952	0.055	−0.113 to 0.219	0.511
Fine	0.082	−0.124 to 0.280	0.422	0.050	−0.160 to 0.255	0.635	0.033	−0.146 to 0.210	0.710	−0.009	−0.255 to 0.239	0.945	−0.017	−0.183 to 0.150	0.836

	5,6‐diHETrE			8,9‐diHETrE			11,12‐diHETrE			14,15‐diHETrE			Total diHETrE		
		*n* = 118			*n* = 199			*n* = 200			*n* = 200			*n* = 200	
VABS‐Ⅱ	*r*	95% CI	*P*	*r*	95% CI	*P*	*r*	95% CI	*P*	*r*	95% CI	*P*	*r*	95% CI	*P*
Communication	0.064	−0.123 to 0.247	0.490	−0.112	−0.251 to 0.031	0.114	−0.118	−0.256 to 0.025	0.096	−0.027	−0.169 to 0.117	0.708	−0.090	−0.230 to 0.053	0.203
Expressive Language	−0.002	−0.188 to 0.184	0.981	−0.110	−0.249 to 0.034	0.122	−0.132	−0.270 to 0.01	0.061	−0.038	−0.180 to 0.105	0.594	−0.107	−0.246 to 0.037	0.132
Receptive Language	0.106	−0.082 to 0.286	0.255	−0.115	−0.254 to 0.029	0.107	−0.075	−0.216 to 0.068	0.290	−0.006	−0.149 to 0.136	0.927	−0.047	−0.189 to 0.096	0.507
Writing	0.080	−0.107 to 0.262	0.387	−0.058	−0.200 to 0.086	0.415	−0.068	−0.209 to 0.075	0.337	−0.022	−0.165 to 0.121	0.752	−0.065	−0.206 to 0.078	0.359
Daily Living Skills	0.118	−0.070 to 0.297	0.204	−0.084	−0.225 to 0.059	0.236	−0.092	−0.232 to 0.052	0.196	−0.057	−0.198 to 0.087	0.426	−0.080	−0.221 to 0.063	0.258
Community	0.060	−0.128 to 0.243	0.520	−0.054	−0.196 to 0.090	0.447	−0.115	−0.254 to 0.028	0.105	−0.042	−0.184 to 0.102	0.556	−0.062	−0.203 to 0.082	0.385
Domestic	0.077	−0.110 to 0.260	0.404	−0.132	−0.270 to 0.011	0.063	−0.093	−0.232 to 0.051	0.191	−0.098	−0.237 to 0.046	0.168	−0.128	−0.266 to 0.015	0.071
Personal	0.149	−0.038 to 0.326	0.108	−0.035	−0.178 to 0.108	0.621	−0.021	−0.163 to 0.122	0.770	−0.009	−0.152 to 0.134	0.90	−0.009	−0.152 to 0.134	0.898
Socialization	0.133	−0.054 to 0.312	0.150	−0.017	−0.160 to 0.126	0.808	−0.158	−0.294 to −0.016	0.025	−0.130	−0.267 to 0.013	0.067	−0.079	−0.219 to 0.064	0.266
Coping Skills	0.072	−0.115 to 0.255	0.436	−0.031	−0.173 to 0.113	0.665	−0.202	−0.335 to −0.061	0.004*	−0.143	−0.280 to 0.0002	0.044	−0.109	−0.248 to 0.035	0.126
Interpersonal	0.103	−0.084 to 0.284	0.265	−0.053	−0.194 to 0.091	0.460	−0.165	−0.301 to −0.023	0.019	−0.151	−0.287 to −0.008	0.033	−0.107	−0.246 to 0.037	0.133
Play and Leisure	0.189	0.003 to 0.362	0.040	−0.001	−0.144 to 0.143	0.993	−0.062	−0.203 to 0.082	0.385	−0.056	−0.197 to 0.087	0.431	−0.016	−0.159 to 0.127	0.820
Motor	0.105	−0.083 to 0.285	0.257	−0.006	−0.205 to 0.079	0.928	−0.064	−0.205 to 0.079	0.367	−0.059	−0.200 to 0.085	0.409	−0.041	−0.183 to 0.102	0.561
Gross	0.069	−0.119 to 0.252	0.458	−0.081	−0.222 to 0.063	0.253	−0.088	−0.228 to 0.055	0.215	−0.057	−0.198 to 0.087	0.426	−0.090	−0.230 to 0.053	0.205
Fine	0.084	−0.104 to 0.266	0.366	0.043	−0.101 to 0.185	0.550	−0.021	−0.164 to 0.122	0.765	−0.042	−0.184 to 0.101	0.552	0.006	−0.137 to 0.148	0.935

	5,6‐diHETrE/5,6‐EET			8,9‐diHETrE/8,9‐EET			11,12‐diHETrE/11,12‐EET			14,15‐diHETrE/14,15‐EET			Total diHETrE/Total EET		
		*n* = 76			*n* = 94			*n* = 128			*n* = 67			*n* = 147	
VABS‐Ⅱ	*r*	95% CI	*P*	*r*	95% CI	*P*	*r*	95% CI	*P*	*r*	95% CI	*P*	*r*	95% CI	*P*
Communication	−0.133	−0.354 to 0.102	0.252	−0.054	−0.259 to 0.156	0.606	−0.022	−0.200 to 0.157	0.805	−0.097	−0.336 to 0.154	0.435	−0.036	−0.202 to 0.131	0.664
Expressive Language	−0.106	−0.330 to 0.129	0.361	−0.098	−0.300 to 0.113	0.350	−0.047	−0.224 to 0.133	0.599	−0.071	−0.312 to 0.179	0.569	−0.079	−0.243 to 0.089	0.340
Receptive Language	−0.242	0.449 to 0.011	0.035	−0.045	−0.251 to 0.165	0.667	−0.038	−0.215 to 0.142	0.671	−0.178	−0.407 to 0.072	0.149	−0.115	−0.276 to 0.053	0.166
Writing	0.027	−0.206 to 0.257	0.816	−0.052	−0.258 to 0.158	0.619	0.028	−0.152 to 0.205	0.757	0.011	−0.237 to 0.257	0.932	0.095	−0.073 to 0.258	0.252
Daily Living Skills	−0.005	−0.236 to 0.228	0.969	−0.112	−0.313 to 0.099	0.282	−0.086	−0.260 to 0.094	0.336	−0.071	−0.313 to 0.179	0.568	−0.040	−0.205 to 0.128	0.632
Community	−0.034	−0.264 to 0.199	0.771	−0.148	−0.346 to 0.062	0.153	−0.077	−0.252 to 0.103	0.388	−0.027	−0.272 to 0.222	0.83	−0.055	−0.220 to 0.112	0.507
Domestic	−0.075	−0.301 to 0.160	0.522	−0.128	−0.328 to 0.083	0.219	−0.023	−0.200 to 0.156	0.799	0.015	−0.233 to 0.261	0.907	−0.100	−0.262 to 0.068	0.227
Personal	0.062	−0.172 to 0.290	0.593	−0.024	−0.231 to 0.186	0.822	−0.052	−0.228 to 0.128	0.561	−0.069	−0.310 to 0.181	0.581	−0.002	−0.169 to 0.165	0.981
Socialization	−0.057	−0.285 to 0.177	0.626	−0.038	−0.245 to 0.171	0.713	−0.105	−0.278 to 0.075	0.239	−0.056	−0.299 to 0.193	0.650	−0.185	0.341 to 0.019	0.025
Coping Skills	−0.131	−0.352 to 0.104	0.260	−0.089	−0.292 to 0.121	0.392	−0.152	−0.201 to 0.156	0.087	−0.151	−0.384 to 0.100	0.222	−0.225	−0.377 to −0.061	0.006*
Interpersonal	−0.119	−0.342 to 0.116	0.305	−0.048	−0.254 to 0.162	0.643	−0.149	−0.319 to 0.031	0.094	0.031	−0.218 to 0.276	0.803	−0.186	−0.342 to −0.020	0.024
Play and Leisure	0.089	−0.146 to 0.315	0.443	0.015	−0.194 to 0.223	0.885	0.010	−0.169 to 0.188	0.911	−0.049	−0.292 to 0.201	0.695	−0.089	−0.252 to 0.079	0.285
Motor	0.117	−0.118 to 0.340	0.314	−0.068	−0.272 to 0.143	0.518	−0.013	−0.191 to 0.166	0.887	−0.071	−0.313 to 0.179	0.566	0.001	−0.166 to 0.168	0.990
Gross	0.065	−0.169 to 0.293	0.574	−0.047	−0.253 to 0.163	0.655	0.062	−0.117 to 0.238	0.484	−0.005	−0.252 to 0.242	0.967	−0.041	−0.206 to 0.127	0.624
Fine	0.078	−0.157 to 0.304	0.505	−0.054	−0.260 to 0.156	0.603	−0.046	−0.223 to 0.134	0.607	−0.073	−0.315 to 0.177	0.555	0.011	−0.155 to 0.178	0.890

Asterisk indicate *P < 0.01, spearman rank correlation coefficient. 95% CI, 95% confidence interval; AA, arachidonic acid; , diHETrE, dihydroxy eicosatrienoic acid; EET, epoxy eicosatrienoic acid; RRB, restricted interests and repetitive behavior; VABS‐II, Vineland Adaptive Behavior Scales, Second Edition.

AA‐derived diols were associated with ASD symptoms and social adaptive functioning; therefore, we also evaluated the activity of sEH, which is upstream of the metabolism of EpFAs to diols and helps catalyze this process. The ratio of eicosanoids (diols/EpFAs), a known proxy marker for *in vivo* sEH activity,^29^ was calculated in this study. No significant relationship was found for ADOS‐2 CSS and subdomain scores in the sEH activities corresponding to LA, AA, EPA, and DHA. This result was also consistent with the sex‐specific validation (Tables 2, 3, 4). In contrast, a significant negative correlation was found for coping skills, the socialization domain of adaptive functioning, with sEH activity levels expressed as total diHETrE/total EET (*r* = −0.225, 95% CI = −0.377 to −0.061, *P* = 0.006) (Table 5). In addition, a significant negative correlation trend with total diHETrE/total EET was confirmed in the socialization domain of Interpersonal (*r* = −0.186, 95% CI = −0.342 to −0.020, *P* = 0.024). This indicates that the adaptive function of socialization is increasingly impaired with increasing total diHETrE/total EET ratio. Insignificant differences were found in the variables based on sex (Table 5). Furthermore, no significant relationship was found between adaptive function and any sEH activities corresponding to LA, EPA, and DHA (data not shown).

## Discussion

**Fig. 2:**
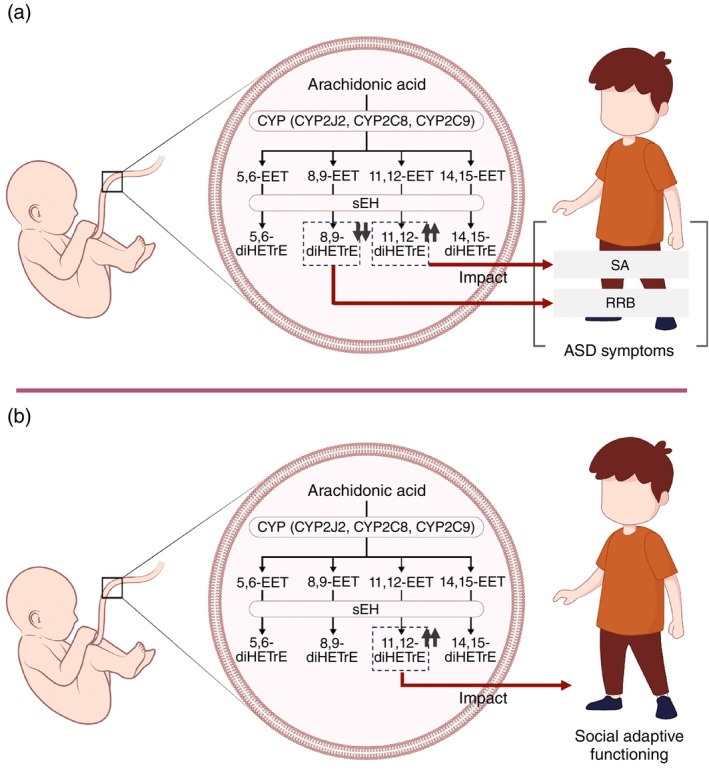
Schematic figure of arachidonic acid (AA) metabolism in neonatal cord blood and its relation to ASD. AA released from phospholipid membranes is metabolized to 5,6‐, 8,9‐, 11,12‐, and 14,15‐EET by CYP epoxygenases, represented by CYP2J2, CYP2C8 and CYP2C9. SEH hydrolyzes four EETs to 5,6‐, 8,9‐, 11,12‐, and 14,15‐diHETrE with very low biological activity. Of these, high levels of 11,12‐diHETrE in cord blood impact subsequent ASD symptoms, particularly SA, in children and are also associated with adaptive functions in sociability. In contrast, 8,9‐diHETrE at low levels impact RRB. (ASD, autism spectrum disorders; CYP, cytochrome P450; diHETrE, dihydroxy eicosatrienoic acid; EET, epoxy eicosatrienoic acid; RRB, repetitive/restrictive behavior; sEH, soluble epoxide hydrolase; SA, social affect).

This study illustrates that the dynamics of AA‐derived diols in cord blood are important in defining the biological context of impaired adaptive functioning and behavioral features of ASD. Specifically, high levels of AA‐derived diols in cord blood, including total diHETrE, 11,12‐diHETrE, and 14,15‐diHETrE, were found to impact ASD symptom severity significantly. At high levels of 11,12‐diHETrE, it was associated with SA disability. Detailed examination of boys and girls separately showed significant associations between AA‐derived diols and ASD symptoms in girls. Furthermore, higher 11,12‐diHETrE levels were associated with impaired adaptive functioning in social domains, such as coping skills. Based on these findings, AA‐derived metabolites in cord blood may influence subsequent neurodevelopment in children, leading to ASD symptoms and adaptive functioning impairment (Fig. 2a,b).

AA is produced in four positional isomers, namely, 5,6‐, 8,9‐, 11,12‐, and 14,15‐EET, by the major CYP epoxygenases, CYP2J2, CYP2C8, and CYP2C9. Each EET is further hydrolyzed by sEH, a product of the *EPHX2* gene, to the corresponding diHETrE (5,6‐, 8,9‐, 11,12‐, and 14,15‐ diHETrE) (Fig. 2).^30^ diHETrE is a metabolite with very low biological activity, but several studies have reported its unique functions. For example, animal models of blood 11,12‐diHETrE reportedly have potent vasodilatory effects and are mediated by the activation of KCa channels.^31^ *In vitro* studies have described 14,15‐diHETrE as an endogenous metabolite involved in lipid metabolism and inflammatory signaling.^32^ In particular, the proinflammatory effects of diHETrE^13^, ^33^ may lead to MIA‐mediated effects on the developing fetal CNS by inflammatory mediators, which is thought to be an aspect of the ASD pathogenesis mechanism.^8^

One interesting aspect of the present study is that among the CYP‐PUFA metabolites, only diHETrE were associated with ASD. In contrast, diHOME and diHETE were not associated with ASD symptoms despite their role as inflammatory mediators.^34^, ^35^ This may be because only diHETrE,^13^ but not diHOME, diHETE, or diDoPE, has been shown to affect the proinflammatory cytokine MCP‐1 in monocyte motility. Indeed, increased MCP‐1 levels in the fetal mouse brain result in ASD‐like behaviors associated with sociality and anxiety, which are ameliorated in the absence of MCP‐1.^36^ Furthermore, this report suggests that IL‐6 production is dependent on MCP‐1,^36^ and IL‐6 is known to be associated with MIA‐mediated ASD‐like behavioral abnormalities.^37^ These findings suggest that diHETrE may influence the development of ASD symptoms and adaptive functions *via* inflammatory cytokines, including MCP‐1 and IL‐6. diHETrE may also have an unknown biological activity, such as endogenous ligands for orphan receptors, which accounts for its association with ASD symptoms.

It bears repeating that this study strongly suggests that diHETrE, which induces inflammation, plays an essential role in the development of a broad spectrum of ASD functions. Because 11,12‐diHETrE was significantly associated with two separate measures of ASD symptoms and adaptive functioning, the association was unlikely to occur by chance. A previous study reported that increased levels of 11,12‐diHETrE occur in the prefrontal cortex of murine male offspring of glyphosate‐exposed pregnant mice, inducing ASD‐like behavior.^18^ However, in humans, reduced cord blood diHETrE has been reported in boys with ASD,^9^ contradicting the present study's results. Examination of the association with AA‐derived metabolites for the ADOS‐2 subdomains SA and RRB revealed that increased SA was associated with increased 11,12‐ and total diHETrE. However, elevated RRB was associated with decreased 8,9‐diHETrE (Table 4). These findings were consistent with those of Che *et al*. (2023)^9^ for RRB and contradicted assumptions about ASD symptom severity and SA. The action of diHETrE like an anti‐inflammatory only in RRB remains unclear. In addition to ASD symptom severity, SA is likely to be affected by diHETrE, a known proinflammatory factor. Validating ASD separately by trait is crucial for elucidating the MIA hypothesis. Importantly, ASD symptoms were more associated with AA‐derived diols (8,9‐, 11,12‐, and total diHETrE) in girls than in boys. Moreover, the association with SA was more prominent in girls. Unlike previous studies, the strong trend in girls in this study may be due to the ratio of boys to girls was nearly equal, and that severity was assessed using the ADOS‐2 CSS rather than diagnosis. In contrast, in adaptive functioning, the association with diHETrE disappeared for both boys and girls when examined separately based on sex. This may be because girls have better adaptive functioning than boys, making the degree of impairment in adaptive functioning less visible. Indeed, girls in the HBC Study scored higher than boys in the communication domain of the VABS‐II, and in the socialization domain, girls were shown to be better in the less adaptive classes.^38^ Taken together, these data suggest that an imbalance in inflammatory mediators, as indicated by high or low diHETrE levels, may affect ASD, which may be more pronounced in girls. Particularly, alterations in diHETrE levels in the fetal brain may reflect neuroinflammation that affects CNS health. Indeed, diHETrE was detected in human cerebrospinal fluid (CSF),^39^ and confirmed in human placental and umbilical cord tissue.^40^ In the CSF of children with ASD, sensitive markers of neuroinflammation such as quinolinic acid and neopterin are decreased, suggesting a typical dysregulated inflammatory response,^41^ and immune response genes are upregulated in the postmortem brains of patients with ASD.^42^ Our findings support these previous studies. However, no previous studies have reported whether diHETrE can cross the blood–brain barrier; thus, more studies are needed.

The role of sEH, upstream of AA‐derived diol production, in ASD development remains to be fully elucidated. In this study, the higher the sEH activity, as indicated by total diHETrE/total EET, the more impaired the adaptive functioning of sociability. However, ASD symptoms were not significant, which may be due to the small sample size. Recent studies have shown that sEH inhibition suppresses the activation of the mitogen‐activated protein kinase and NF‐κB signaling pathways and mediates the activation of the nuclear factor E2‐related factor 2 (Nrf2) signaling pathway, thereby modulating inflammatory responses and oxidative stress.^43^ Notably, reports revealed decreased expression of the Nrf2 gene, *NFE2L2*, in the frontal cortex of individuals with ASD^44^ and potential links between activation of NF‐κB and ASD etiology.^45^ These observations highlight the role of sEH activity in ASD. Thus, these findings suggest that increased sEH activity promotes metabolism to diHETrE, leading to inflammation and oxidative stress responses, causing ASD.

Meanwhile, based on our hypothesis, it appears intuitive that EET, the precursor of diHETrE, would be reduced by sEH and associated with ASD. Interestingly, no association was found between EET and ASD symptoms or adaptive function in this study. The reason is unclear; however, the various enzymes (EPHX1, EPHX3, and EPHX4)^46^, ^47^ involved in the metabolic process from EET to diHETrE are complexly affected depending on the organism's conditions. In addition, the effect of genotypes on diHETrE and EET levels in cord blood cannot be excluded. Previous studies have reported that the promoter polymorphism G‐50T of CYP2J2 reduces diHETrE levels by decreasing the transcription of *CYP2J2*,^48^ and a human single‐nucleotide polymorphism in *EPHX2*, Arg287Gln, reduces sEH activity in cells.^49^ Stratification according to allelic differences and exhaustive examination, including hydrolytic enzymes may reveal more detailed mechanisms of the relationship between diHETrE and ASD, which is an important future perspective.

This study has several limitations. First, it was limited in its generalizability because the results were obtained from a specific region and a single population. Therefore, the proportion of children with ASD in the sample of this study was higher than expected (Table 1). Second, although cord blood samples taken immediately after birth were used to quantify CYP‐PUFA metabolites and to assess ASD symptoms and adaptive functioning in children aged 6 years, these are only partial assessments of the developmental process. Importantly, we did not quantify CYP‐PUFA metabolites at age 6, making it impossible to describe the association between the inflammatory state caused by postnatal diols and ASD symptoms and adaptive functioning. Verification of changes along the developmental trajectory is needed to understand the impact of the prenatal and postnatal inflammatory milieu. Third, many EpFA samples were below the LOD compared with diols. The probable explanation is that EpFA is an intermediate metabolite in fatty acid metabolism that easily becomes the end product of diol. EpFA is structurally more unstable than diol, making it more susceptible to hydrolysis and redox reactions. Fourth, the statistical significance level was defined as 0.01; however, multiple testing may have introduced a type I error. Given that the ADOS‐2 and VABS‐II scales clearly showed significant relationships only for diHETrE, we avoided using multiple comparisons because it might miss truly significant results.^50^ The issue of multiplicity should be considered when examining these results.

A strength of this study, in contrast, is its focus on the severity of ASD symptoms rather than on the presence or absence of an ASD diagnosis. Based on the CSS score, 34 (17%) scored 8–10 for severe ASD symptoms, and 39 (19.5%) scored 5–7 for moderate ASD symptoms. Previous studies have shown that many children diagnosed with ASD fall within the 7–9 range on the ADOS‐2 CSS.^51^, ^52^ However, an ASD diagnosis is not necessarily associated with symptom severity, as children without ASD diagnosis may also be rated as having moderate or mild ASD symptoms based on the ADOS‐2 CSS.^51^, ^52^ We intentionally avoided comparison of those with and without ASD based on the clinical assessment. Rather, we successfully showed the biological gradient of the influence of diol concentration *in utero* on the later occurrence of autistic phenotypes.

In conclusion, levels of diHETrE, an AA‐derived diol, in cord blood at birth significantly impacted subsequent ASD symptoms in children and were associated with impaired adaptive functioning. The relationship between ASD symptoms and diHETrE was more specific for girls than for boys. These results suggest that the dynamics of diHETrE involved in inflammatory cytokine‐mediated MIA are important in the developmental trajectory of children after birth. This study is the first to report the role of diol metabolites, particularly in relation to neurodevelopment, and thus provides important insights into the role of diHETrE and ASD pathophysiology.

## Disclosure statement

Authors THi, KJT, and HM are now planning patents related to the data reported in this manuscript. Author TO‐N is employed with Lipidome Lab Co., Ltd. The remaining authors declare that the research was conducted without any commercial or financial relationships that could be construed as a potential conflict of interest.

## Author contributions

HM conceived and organized this study. THi analyzed the data and drafted the manuscript. THa, AO, CN, KJT, and TN provided investigation and resources. NU and TN analyzed and interpreted the data. TO‐N performed the LC–MS/MS analysis. KJT organized the HBC Study and supervised the study. All the authors contributed to the discussion of the results and the creation of this manuscript.

## Supporting information


**Table S1.** Comparison of the characteristics of subset and HBC Study.

**Table S2.** Correlation of VABS‐II with arachidonic acid‐derived metabolites separately based on sex.
